# The Innate Immune Response in HIV/AIDS Septic Shock Patients: A Comparative Study

**DOI:** 10.1371/journal.pone.0068730

**Published:** 2013-07-11

**Authors:** Rodrigo T. Amancio, Andre M. Japiassu, Rachel N. Gomes, Emersom C. Mesquita, Edson F. Assis, Denise M. Medeiros, Beatriz Grinsztejn, Patrícia T. Bozza, Hugo C. Castro-Faria, Fernando A. Bozza

**Affiliations:** 1 Laboratório de Medicina Intensiva, Instituto de Pesquisa Clinica Evandro Chagas (IPEC), Rio de Janeiro, RJ, Brazil; 2 Laboratório de HIV, Instituto de Pesquisa Clinica Evandro Chagas (IPEC), Rio de Janeiro, RJ, Brazil; 3 Laboratório de Imunofarmacologia, Instituto Oswaldo Cruz (IOC), Fundação Oswaldo Cruz (FIOCRUZ), Rio de Janeiro, RJ, Brazil; 4 Instituto d’Or de Pesquisa e Ensino (IDOR), Rio de Janeiro, RJ, Brazil; D'or Institute of Research and Education, Brazil

## Abstract

**Introduction:**

In recent years, the incidence of sepsis has increased in critically ill HIV/AIDS patients, and the presence of severe sepsis emerged as a major determinant of outcomes in this population. The inflammatory response and deregulated cytokine production play key roles in the pathophysiology of sepsis; however, these mechanisms have not been fully characterized in HIV/AIDS septic patients.

**Methods:**

We conducted a prospective cohort study that included HIV/AIDS and non-HIV patients with septic shock. We measured clinical parameters and biomarkers (C-reactive protein and cytokine levels) on the first day of septic shock and compared these parameters between HIV/AIDS and non-HIV patients.

**Results:**

We included 30 HIV/AIDS septic shock patients and 30 non-HIV septic shock patients. The HIV/AIDS patients presented low CD4 cell counts (72 [7-268] cells/mm^3^), and 17 (57%) patients were on HAART before hospital admission. Both groups were similar according to the acute severity scores and hospital mortality. The IL-6, IL-10 and G-CSF levels were associated with hospital mortality in the HIV/AIDS septic group; however, the CRP levels and the surrogates of innate immune activation (cytokines) were similar among HIV/AIDS and non-HIV septic patients. Age (odds ratio 1.05, CI 95% 1.02-1.09, *p*=0.002) and the IL-6 levels (odds ratio 1.00, CI 95% 1.00-1.01, *p*=0.05) were independent risk factors for hospital mortality.

**Conclusions:**

IL-6, IL-10 and G-CSF are biomarkers that can be used to predict prognosis and outcomes in HIV/AIDS septic patients. Although HIV/AIDS patients are immunocompromised, an innate immune response can be activated in these patients, which is similar to that in the non-HIV septic population. In addition, age and the IL-6 levels are independent risk factors for hospital mortality irrespective of HIV/AIDS disease.

## Introduction

A significant improvement in the prognosis of the HIV/AIDS population was observed after the introduction and widespread use of highly active antiretroviral therapy (HAART) [1]. During HAART era, the causes of hospitalization and death among HIV/AIDS patients have changed, with a relative increase on noninfectious conditions and non–AIDS-defining infections [2-5].

In recent years, the incidence of sepsis in the HIV/AIDS population has increased as a cause of Intensive Care Unit (ICU) admission [6]. Epidemiological studies have demonstrated that approximately 1-10% of septic patients are composed of HIV/AIDS patients with regional variations due to local differences in HIV prevalence [7-12]. The presence of sepsis and severe sepsis is a major determinant of outcomes in critically ill HIV/AIDS patients, which impacts short- and long-term survival [13,14].

An excessive and deregulated inflammatory response plays a key role in the development of multiple organ dysfunctions in sepsis [15]. Cytokine measurements have been used to identify cytokine patterns that are associated with disease severity, organ dysfunction and prognosis in septic patients [16]. Acute and chronic HIV infection triggers the release of inflammatory mediators and cytokines. In patients with advanced HIV disease, elevated C-reactive protein (CRP), IL-6 and D-dimer levels before initiating HAART are strongly associated with early mortality [17]. These inflammatory mediators have previously been evaluated in HIV/AIDS patients to stratify cardiovascular risk, disease progression, infection diagnosis and prognosis [18-20]. However, inflammatory response patterns during acute severe infection have not been systematically evaluated in HIV/AIDS patients. Therefore, the aim of this study was to evaluate the clinical and laboratorial differences between HIV/AIDS septic shock patients and non-HIV septic shock patients with focus on the surrogates of the innate immune response. Similar to the non-HIV patients, we observed that an innate immune response was triggered in the HIV/AIDS patients despite a cellular immunodeficiency due to HIV infection.

## Materials and Methods

### Ethics Statement

The study was conducted in accordance with the Declaration of Helsinki. Written informed consent was obtained from all patients or their surrogate decision makers, prior to any study-related procedure. The Institutional Review Board approved study protocol (Instituto de Pesquisa Clínica Evandro Chagas, CAAE 0014.0.009.000-08). Participants who refused to participate or were excluded by exclusion criteria were not harmed by not participating in the study.

### Design and setting

We conducted a prospective multicenter cohort study of HIV/AIDS patients at the ICU of the Instituto de Pesquisa Clínica Evandro Chagas (IPEC), Fundação Oswaldo Cruz (FIOCRUZ) in Rio de Janeiro, Brazil. This institution has provided care to HIV/AIDS patients since 1986. There are currently more than 2,000 adult patients who are actively followed up at the HIV/AIDS Clinic. We admit approximately 60-70 critically ill HIV/AIDS patients per year in the ICU at this institution. All of the consecutive HIV/AIDS adult patients (≥ 18 years of age) who were admitted to the ICU with a diagnosis of septic shock from March 2008 to December 2010 were included. Non-HIV septic shock non-HIV patients were included from three different hospitals, also located in Rio de Janeiro, Brazil: Clementino Fraga Filho University Hospital, Hospital Quinta D’Or and Casa de Saúde São José. These three different hospitals resemble each other because they are tertiary hospitals, with mixed medical-surgical ICUs, with an average of 30 beds each. HIV and non-HIV patients were enrolled according to similar criteria for septic shock.

### Definitions, selection of participants and data collection

The sepsis definitions were based on the ACCP/SCCM Consensus Conference (Bone, 1992). Sepsis was present when there was a presumed or confirmed infection according to the SIRS criteria. The severity of illness was assessed by calculating the Simplified Acute Physiology Score (SAPS II) during the first 24 hours of infection [21] and the Sequential Organ Failure Assessment (SOFA) score on the first and third day after sepsis diagnosis [22]. Acute organ dysfunction was categorized as at least a 1-point classification in the SOFA score.

Patients were eligible for inclusion when they fulfilled the criteria for a systemic inflammatory response, had an obvious source of infection, and shock diagnosis within the previous 48 hours. Shock was defined as persistent hypotension after fluid resuscitation and the need for at least one vasopressor agent. Patients were excluded when death occurred within 6 hours of admission or when patients were under 18 years of age. In addition, pregnancy, patients with advanced cancer, a hematological malignancy or do-not-resuscitate (DNR) orders were excluded.

Demographic, clinical and laboratory data were collected using standardized case report forms on the first day of a sepsis diagnosis. Additionally, we collected the following data related to HIV infection: time since diagnosis of HIV infection, CD4 count and HAART use. The most recent CD4 count before the inclusion date was collected. The ICU and hospital mortality rates were assessed.

The appropriate biological material was sampled from suspected sites of infection and collected as recommended by the Centers for Disease Control and Prevention (CDC) [23]. Microbiological data were collected and analyzed according to the local Infection Control Committee.

Blood samples were obtained using an arterial line or a peripheral vein to assess CRP and cytokine levels on the first day of septic shock diagnosis.

### C-reactive protein (CRP) measurement

CRP levels were measured using the nephelometric method with a Minineph commercial kit (The Binding Site Group, Ltd., Birmingham, UK) according to manufacturer’s orientations. Briefly, the determination of soluble antigen concentration by nephelometric methods involves a reaction with the antibody bound to a latex particle to form insoluble complexes. The approximate measuring range is 3.5–112 mg/L at a sample dilution of 1/40. The sensitivity limit is 0.44mg/L when using a 1/5 sample dilution. Sample concentrations higher than the stated range may result in antigen excess, which can give misleading results, and than samples should be retested at a 1/440 dilution.

### Multiplex cytokine assay

A multiplex cytokine kit (IL-1β, IL-2, IL-4, IL-5, IL-6, IL-7, IL-8, IL-10, IL-12, IL-13, IL-17, IFN-γ, granulocyte colony-stimulating factor [G-CSF], granulocyte-macrophage colony-stimulating factor [GM-CSF], monocyte chemoattractant protein [MCP]-1, macrophage inflammatory protein-1 and tumor necrosis factor [TNF]-α) was obtained, and the assay was performed according to the manufacturer’s instructions (Bio-Rad, Hercules, CA, USA). We conducted this assay according to previous experience [16]. The data analyses for all of the assays were performed using the Bio-Plex Manager software. Only cytokines with detectable levels above 90% in all of the samples were considered for the analysis: IL1-β, IL-4, IL-6, IL-7, IL-8, IL-10, IL-12, G-CSF, MCP-1, MIP-1β and TNF-α.

### Statistical analysis

Statistical analyses were performed using GraphPad Prism, version 5.0 for Windows (GraphPad Software, San Diego, CA, USA) or the Statistical Package for the Social Sciences (SPSS 16.0, SPSS Inc., Chicago, IL, USA). The numerical demographic variables were expressed as the median and the interquartile range (IQ 25-75%), except cytokine and CRP values, which were expressed as the median, minimum and maximum values. All of the numerical variables were tested for a normality distribution using the Kolmogorov-Smirnov test. We applied the logarithmic transformation when large variability and nonparametric distribution of cytokines levels were observed. We compared the continuous variables using a *t*-test (parametric distribution) and the Mann–Whitney U test (non-parametric distribution). The categorical variables were compared using the chi-squared test and Fisher’s exact test. We compared the mean cytokine values of the survivors and the non-survivors on the first day of sepsis diagnosis. We compared the clinical and laboratorial variables within the HIV group according to the hospital outcomes. These same variables were compared among the HIV and non-HIV patients. A multivariate analysis was performed by logistic regression to determine which factors were associated with hospital mortality. The data from all of the patients (HIV/AIDS and non-HIV) with a *p* value lower than 0.2 in the univariate analysis were included in the multivariate analysis. The objective of the multivariate analysis was to ascertain if the presence of HIV/AIDS could influence hospital outcomes.

## Results

### Cytokine concentrations

When data from all 1,020 assays were analyzed, the multiplex cytokine system was able to detect plasma cytokines in 820 (80%) assays. The concentrations of TNF-alpha, IL-1beta, IL-4, IL-6, IL-7, IL-8, IL-10, IL-12, IL-13, G-CSF, IFN-gamma, MCP-1 and MIP-1beta were detectable in more than 90% of individual assays, and only four cytokines (IL-2, IL-5, IL-17 and GM-CSF) presented detection below 50% of the samples (File S1). There was no difference in the rate of cytokine detection between HIV and non-HIV septic shock patients and between survivors and non-survivors (File S1).

### Clinical characteristics of the HIV/AIDS and non-HIV septic shock patients

We included 30 HIV/AIDS septic shock patients. The demographic and clinical characteristics are described in Table 1. The patients were predominantly young (median age 32 years) with low CD4 cell counts (median 72 cells/mm^3^), and the median time that had elapsed since a HIV diagnosis was 12.5 months. Among the 30 HIV/AIDS patients, 17 (57%) were already using HAART before hospital admission. Thirteen patients were HAART-naïve, and 11 of these patients started the therapy during their ICU stay.

**Table 1:** Characteristics of HIV/AIDS and non-HIV septic patients. The results were expressed as the median and the interquartile interval.

	HIV/AIDS Patients(N=30)	Non-HIV Patients(N=30)		*p* value
Age (years)	32 (26-43)	66 (46-78)		<0.001
Gender (male)	22 (73%)	18 (60%)		0.41
SAPS II score (points)	55 [47-58]	55 [44-63]		0.63
CD4 cell count (per mm^3^)	72 (16-190)	-		-
Viral load (x 10^3^ copies/mm^3^	33.0 (2.9-434.2)	-		-
Length of time since AIDS diagnosis (months)	12.5 (1-86)	-		-
HAART use	17 (57%)	-		-
Site of infection				0.01
Lung	23 (77%)	18 (60%)		
Abdomen	-	8 (28%)		
Central line infection	3 (10%)	3 (10%)		
Central nervous system	3 (10%)	-		
Urinary tract	1 (3%)	1 (3%)		
SAPS II score (points)	55 [47-58]	55 [44-63]		0.63
SOFA score on day 1 (points)	8 [6-10.5]	10 [9-13]		0.11
SOFA score on day 3 (points)	9 (5-12)	8 (6-10)		0.66
Hospital mortality	15 (50%)	18 (60%)		0.60

To better understand the HIV/AIDS population with severe sepsis, we compared these patients with a group of 30 non-HIV septic shock patients (Table 1). The HIV/AIDS patients were younger than the non-HIV control patients (median age 32 *vs.* 66 years; *p*<0.001). Pneumonia was the most common site of infection for both groups (77% of HIV/AIDS patients and 60% of non-HIV patients). There was a higher incidence of central nervous system infections in the HIV/AIDS septic patients (10%), whereas a higher incidence of abdominal infections (28%) was found in the non-HIV group. The cultures were positive in 20 (67%) HIV/AIDS septic patients and in 21 (70%) non-HIV patients (*p*=0.9). There was a higher incidence of Gram-positive infections in the non-HIV septic patients (13% of the HIV/AIDS patients vs. 37% of the non-HIV patients, *p*=0.07), whereas a higher incidence of fungal infections was found in the HIV/AIDS patients (20% of the HIV/AIDS patients vs. 0% of the non-HIV patients, *p*=0.02). Gram-negative infections occurred in a similar frequency in both groups (33% of the HIV/AIDS patients vs. 53% of the non-HIV patients, *p*=0.19) (see the Supplementary electronic material – Table S1 in File S1). The presence of bacteremia was confirmed in 33% of the HIV/AIDS patients and in 16% of the non-HIV patients (*p*=0.77). We found no significant differences between the mean SAPS II scores (55 points [range 47-58] for the HIV patients vs. 55 points [range 44-63] for the non-HIV patients) and the SOFA scores on day 1 (8 points [range 6-10.5] for the HIV patients vs. 10 points [range 9-13] for the non-HIV patients). The cumulative incidence of acute organ dysfunction on the first 3 days after sepsis diagnosis was similar between the HIV and non-HIV septic patients (respectively, cardiovascular 81% vs. 97%; respiratory 77% vs. 88%; renal 73% vs. 71%; hematological 59% vs. 63%; hepatic 41% vs. 38%; neurological 18% vs. 17%; all of the *p* values were non-significant) (Table S2 in File S1). The in-hospital mortality did not vary between the groups (50% of the HIV patients *vs.* 60% of the non-HIV patients, *p*=0.6) (Table 1).

### Biomarkers of outcomes in HIV/AIDS septic shock patients

**Figure 1:**
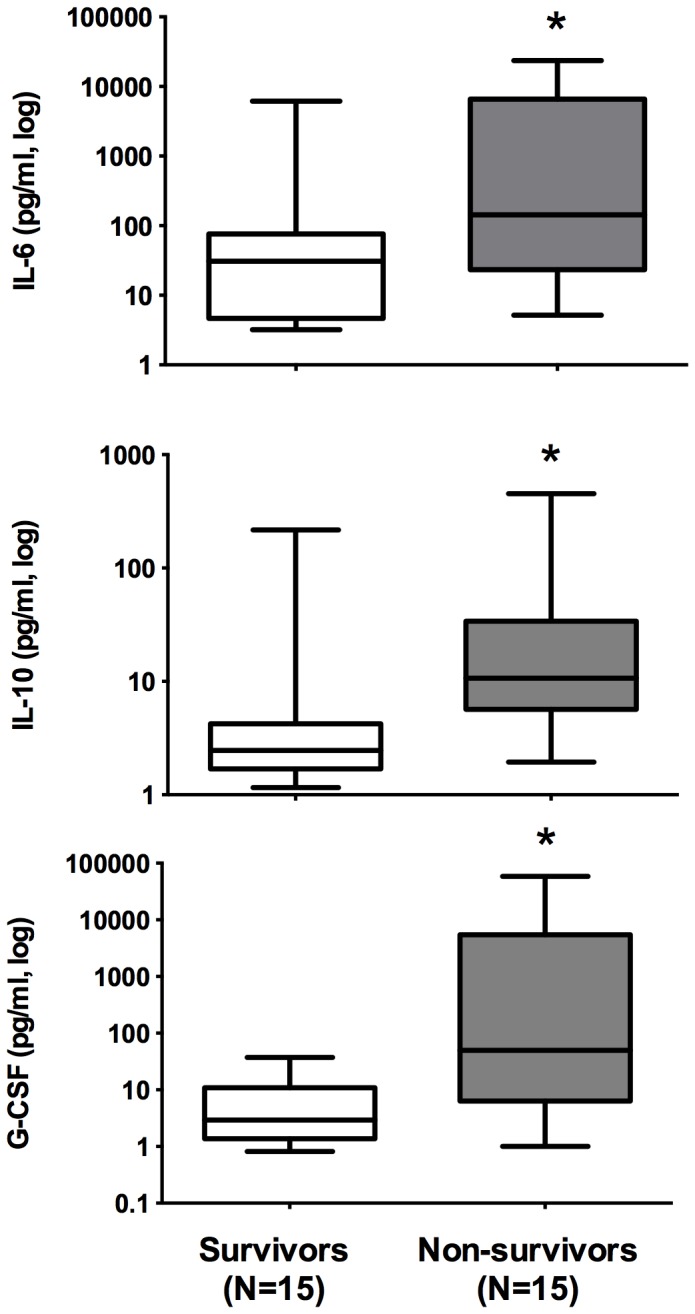
Biomarkers in HIV/AIDS septic patients on the first day of sepsis diagnosis: comparison of the IL-6, IL-10, and G-CSF plasma levels on the first day of sepsis. (*) *p*<0.05.

To identify possible biomarkers of severity and prognosis in the HIV/AIDS septic patients, we compared the inflammatory mediator levels of the survivors and non-survivors. The median log IL-6, (1.49 [0.5-3.7] pg/ml vs. 2.1 [0.7-4.3] pg/ml; *p*=0.03), log IL-10 (0.3 [0.1-2.3] pg/ml vs. 1.0 [0.2-2.6] pg/ml; *P*=0.004) and log G-CSF (0.4 [0.0-1.5] pg/ml vs. 1.6 [0.0-4.7] pg/ml; *p*=0.002) levels were higher in the non-survivors than in the survivors (Figure 1). The area under ROC curve was 0.73 (CI95% 0.55-0.91) for IL-6, 0.80 (0.64-0.97) for IL-10 and 0.82 (0.66-0.97) for G-CSF levels. In addition, we compared the median CRP levels of the survivors and the non-survivors (82.6 [72.0-93.3] mg/L vs. 102.2 [78.3-276.5] mg/L; *p*=0.26) and found no significant differences. These results suggest that IL-6, IL-10 and G-CSF are possible biomarkers of prognosis in HIV/AIDS septic patients.

### Patterns of the innate immune response in HIV/AIDS and non-HIV septic patients

HIV/AIDS septic patients presented lower leucocyte counts than non-HIV patients (6,450 [4,473-11,425] cells/mm^3^ in the HIV/AIDS patients *vs.* 13,100 [7,600-19,400] cells/mm^3^ in the non-HIV patients, *p*=0.007) on the first day of septic shock. We found no significant differences between the CRP levels in the HIV/AIDS septic patients and in the non-HIV septic patients (91.8 [72.3-257.9] mg/L in the HIV/AIDS patients versus 222 [103-350] mg/L in the non-HIV patients, *p*=0.22). The innate immune activation in the HIV/AIDS and non-HIV septic patients was compared, and we found no significant differences in the cytokine levels (Table 2). The HIV/AIDS non-surviving patients presented significantly higher levels of IL-6, IL-10 and G-CSF when compared with the survivors; however, the cytokine levels were similar between the HIV and non-HIV septic patients. These results suggest that an innate immune response was triggered in HIV/AIDS septic patients with similar cytokine patterns as those in non-HIV septic patients despite a cellular immunodeficiency and chronic immune activation.

**Table 2:** Plasma cytokine concentrations on the first day of a sepsis diagnosis in HIV/AIDS and non-HIV septic patients.

Cytokine	HIV/AIDS PatientsN=30	Non-HIV PatientsN=30	*p* value
IL1-β	1.2 (0.8-17.9)	1.5 (0.8-51.2)	0.81
IL-4	0.2 (0.0-4.5)	0.3 (0.0-5.9)	0.23
IL-6*	1.81 (0.5-4.3)	2.37 (0.4-4.1)	0.19
IL-7	3.7 (1.8-15.5)	2.7 (1.2-169.8)	0.23
IL-8	21.6 (1.8-7,260)	27.5 (4.4-532.2)	0.76
IL-10*	0.68 (0.1-2.6)	0.98 (0.1-2.5)	0.45
IL-12	2.8 (2.1-17.9)	2.9 (1.9-211.1)	0.67
G-CSF*	0.89 (0.0-4.7)	1.22 (0.1-4.2)	0.12
MCP-1	137.9 (3.4-4,494)	148.2 (9.0-1,213)	0.67
MIP-1β	53.4 (7.8-1,239)	39.4 (15.4-341.6)	0.55
TNF-α	9.0 (5.9-102.4)	11.0 (5.0-69.4)	0.57

The values were expressed in pg/ml and as the median, maximum and minimum values, * Logarithmic transformation of IL-6, IL-10 and G-CSF concentrations

In addition, we analyzed the independent risk factors that were associated with hospital mortality, including HIV/AIDS diagnosis. Age, comorbidities, the SOFA score, and the IL-6 levels were selected with a *p*-value lower than 0.2 in the univariate analysis. Age (odds ratio 1.05, CI 95% 1.02-1.09, *p*=0.002) and the IL-6 levels (odds ratio 1.00, CI 95% 1.00-1.01, *p*=0.05) were significantly associated with hospital mortality (Table 3).

**Table 3:** Multivariate analysis of hospital mortality among all 60 septic shock patients.

Risk factors	Odds ratio (confidence interval of 95%)		Wald test	*P* value
Age	1.05 [1.02-1.09]		9.87	0.002
Severe comorbidity	-		-	0.17
SOFA score	-		-	0.70
Interleukin-6	1.00 [1.00-1.01]		3.76	0.05

## Discussion

Severe sepsis is an independent risk factor that is associated with short- and long-term mortality in critically ill HIV/AIDS patients [13]. Over the last 3 decades sepsis has become a more frequent cause of admission of HIV/AIDS patients at ICU. A shift towards common bacterial infections rather than opportunistic infections has been observed as a cause of sepsis and mortality in these patients [3,6]. Generalized immune activation has long been detected in AIDS patients and in disease pathogenesis; however, it is unclear how retroviral infections impact the innate immune response and whether these effects influence the outcomes of severe bacterial infections and sepsis.

In this study, we demonstrated that septic shock leads to high levels of inflammatory mediators in HIV/AIDS patients, which were similar to those observed in non-HIV septic shock patients. Higher levels of IL-6, IL-10 and G-CSF were associated with hospital mortality among HIV/AIDS septic patients. In addition to the similar severity of illness (SAPS II and SOFA scores) and hospital mortality, HIV/AIDS septic patients responded to acute infections with the same degree of innate immune response activation as non-HIV septic patients. Age and the IL-6 levels were independent risk factors that were associated with hospital outcomes, and HIV disease was not associated with mortality among the septic shock patients in this study.

The comparison of septic shock patients with and without HIV/AIDS revealed a predominantly young HIV/AIDS population, that were admitted with infections related to low CD4 levels; however, pneumonia was the most common infection in the HIV/AIDS and non-HIV patients. The severity scores (SAPS II and SOFA) and the hospital mortality rates were similar between these two populations. The incidence of organ dysfunction that was associated with sepsis was similar between both groups, which has been described in other studies [24,25]. The mortality rate was similar to those in other studies of HIV/AIDS patients with severe infections [13,25,26].

The etiological agents of the infections that caused severe sepsis in the HIV/AIDS and non-HIV patients were different. The HIV/AIDS septic patients exhibited infections due to bacteria, including *Pseudomonas* sp., *Klebsiella* sp., *Mycobacterium tuberculosis*, *Histoplasma capsulatum* and *Cryptococcus neoformans*, and this pattern was similar to those in other studies of sepsis and severe infections in the HIV population [27,28]. Although these discrepancies in the etiology of infection could lead to variable levels of inflammatory biomarkers [29], our results were similar between the HIV and non-HIV patients according to the responses of the innate immune system.

Sepsis has been described as an excessive and deregulated inflammatory response to infection, and cytokines release has a critical role in the pathogenesis of this syndrome. Elevated levels of pro-inflammatory cytokines, including IL-6, are able to induce CRP secretion, an acute phase protein synthesized by the liver [30]. Acute phase proteins are useful biomarkers for the management of systemic inflammation, as cardiovascular, infectious or autoimmune diseases [31,32]. Additionally, CRP concentrations can be used as surrogates for the clinical response to antibiotics and the reduction in bacterial load is sepsis [33]. We observed a significant increase in CRP concentration in the HIV/AIDS septic patients. Our data suggest that systemic inflammatory response can be induced in HIV patients with septic shock, which is similar in non-HIV patients. Póvoa and colleagues (2011) observed that the CRP levels were increased in severely immunosuppressed cancer patients with sepsis, and CRP course was alike from the presence or absence of neutropenia [34]. If we take these evidences together, it seems that immunosuppression has no major effect on the acute phase response to severe infections. Besides, CRP levels present good correlation with IL-6 levels, suggesting that they vary from patient to patient, regardless of the comorbidities. We suggest that the CRP can also be a useful biomarker of infection in HIV/AIDS septic patients similar to non-HIV septic patients.

Generalized immune activation has long been recognized as a key feature of AIDS pathogenesis. Elevated levels of the T-cell activation marker CD38 were observed in the first AIDS-associated *Pneumocystis* *pneumoniae* cases [35]. The depletion of CD4 cells from gut-associated lymphoid tissue in chronic HIV-infected patients can contribute to microbial translocation, and LPS-induced monocyte activation can promote a persistent inflammatory state, which leads to elevated cytokine levels due to the innate immune system response [36]. High plasmatic levels of monocyte activation markers can be used to predict early mortality in HIV/AIDS patients (SMART). IL-6 is elevated in non-HIV septic patients and is considered one of the best markers of severity/mortality when compared with IL-1 and TNF [37]. IL-6 is a good marker of hospital outcomes in non-HIV septic patients [38]. The IL-6 levels were higher in the non-survivor HIV/AIDS septic patients when compared with the HIV/AIDS survivors. Because the IL-6 levels were similar in the HIV/AIDS and non-HIV septic patients, our data suggest that IL-6 can be a predictor of poor outcomes in the HIV/AIDS septic population. In addition, the IL-10 and G-CSF levels were higher in the HIV/AIDS non-survivors in our study. We applied a multiplex assay for several serum cytokines to identify potential differences in the innate immunological responses. Several cytokines have been associated with a poor prognosis in severely ill patients, particularly septic patients. IL-6 and IL-10 are biomarkers that were associated with hospital mortality in a cohort of general critically ill patients [39]. Additionally, elevated G-CSF concentrations was associated with mortality in acute lung injury patients without AIDS, and these levels could be used to predict early mortality in severe sepsis patients when compared with other cytokine levels in a multiplex assay in a study of a mixed group of septic patients [16,40]. In our study, three cytokines (IL-6, IL-10 and G-CSF) were associated with hospital mortality in HIV/AIDS septic patients. These three biomarkers presented a high performance for the prognosis prediction after ROC analysis; however, the levels of these biomarkers did not vary among the non-HIV septic population. When we analyzed the profile of the cytokines detected in both groups by the multiplex cytokine assay, we observed a predominance of proinflammatory mediators, such as TNF-alpha, IL-1, IL-6, IL-8, and IL-13, while there was low detection of four cytokines (IL-2, IL-5, IL-17 and GM-CSF) with anti-inflammatory action or with compartmental functions, which was expected by biological reasons [41]. In summary, the patterns of elevated innate immune system biomarkers are comparable between HIV/AIDS and non-HIV septic patients.

Our study has several limitations. First, the relatively small sample size limits the subtle associations that were found between hospital outcomes and the HIV/AIDS group and between HIV/AIDS and non-HIV patients. Second, we could not match for age in the HIV/AIDS and non-HIV septic groups. The rate of older patients who are admitted to ICUs worldwide has increased, which affected the three tertiary hospitals that included non-HIV patients; therefore, a small number of young non-HIV septic patients were included [42]. This age limitation was observed in a study during the pre-HAART era [24]; however, this limitation can be overcome by recent data that suggests AIDS leads to a similar immunological impairment that occurs in old age, such as a reduced T cell repertoire, a low naïve/memory T cell ratio and increased IL-6 levels [43]. Another possible limitation is that patients with renal failure and replacement therapy (RRT) on day 1 of severe sepsis (23%) were included in the study. The serum levels of cytokines could be lowered by hemodialysis, although we did not collect blood samples during the procedure. Even though, patients on RRT presented higher serum levels of IL-10, G-CSF and CRP than patients without RRT (data not shown). This data should be interpreted with caution, as our study was not designed to evaluate the effect of renal replacement therapy on serum levels of cytokines. Finally, the use of multiple cytokines panels as biomarkers in the clinical setting is controversial, especially due to a large inter-individual variability and their kinetic oscillation [44]. This variability can challenge the estimation of the power and the sample size of the study. We recalculated the sample size of our study based on the variance of the observed cytokines levels, and our sample was deemed adequate, and the study’s power remained higher than 80%. However, small differences in the cytokine levels between HIV and non-HIV septic shock patients could be underestimated due to their large variance.

We believe that our results can change the current view of HIV/AIDS patients who are generally excluded from studies of new therapies for severe sepsis and septic shock. Mrus et al. demonstrated a similar mortality among HIV/AIDS septic patients and other immunocompromised septic populations, such as cancer and cirrhotic patients. However, septic patients with HIV/AIDS had a lower chance of ICU admission [45]. Our results indicate that there are no discrepancies in the immune response to severe infections or in the prognosis of HIV patients.

## Supporting Information

File S1 Figure S1, Frequency of cytokine detection in the multiplex assay (N=60 patients). Table S1, Number of samples detected. Comparison among HIV and non-HIV patients. Table S2, Number of samples detected. Comparison among survivors and nonsurvivors. Table S3, Microbiology of HIV/AIDS and non-HIV septic patients. Table S4, Frequency of organ dysfunction according to Sequential Organ Failure Assessment (SOFA) score for HIV/AIDS and non-HIV septic shock patients at the first day after the diagnosis of sepsis (p=NS for all of the comparisons).
(PDF)
