# Supplementary material for: The Innate Immune Response in HIV/AIDS Septic Shock Patients: A Comparative Study
Source: PLoS One. 2013 Jul 11;8(7):e68730. doi: 10.1371/journal.pone.0068730 (PMC3708901; doi:10.1371/journal.pone.0068730)
Supplement: File S1 — Figure S1, Frequency of cytokine detection in the multiplex assay (N=60 patients). Table S1, Number of samples detected. Comparison among HIV and non-HIV patients. Table S2, Number of samples detected. Comparison among survivors and nonsurvivors. Table S3, Microbiology of HIV/AIDS and non-HIV septic patients. Table S4, Frequency of organ dysfunction according to Sequential Organ Failure Assessment (SOFA) score for HIV/AIDS and non-HIV septic shock patients at the first day after the diagnosis of sepsis (p=NS for all of the comparisons). (PDF) [file pone.0068730.s001.pdf]

Supplementary electronic material – Supporting Information

Figure S1. Frequency of cytokine detection in the multiplex assay (N=60 patients).

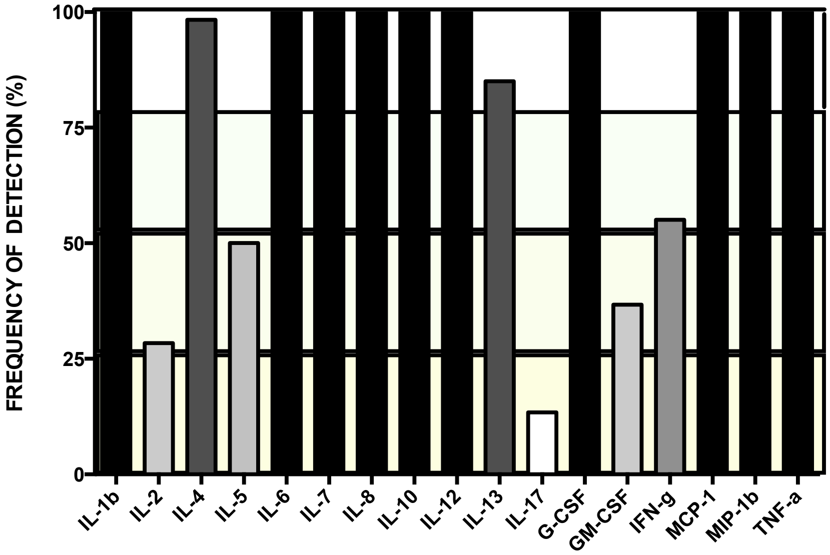

Table S1 – Number of samples detected. Comparison among HIV and non-HIV patients:

Cytokine	HIV (N=30)	Non-HIV (N=30)	p
IL-b	30	30	1.0
IL-2	9	8	0.9
IL-4	29	30	0.9
IL-5	13	17	0.44
IL-6	30	30	1.0
IL-7	30	30	1.0
IL-8	30	30	1.0
IL-10	30	30	1.0
IL-12	30	30	1.0
IL-13	27	24	0.9
IL-17	5	3	0.9
G-SCF	30	30	1.0
GM-CSF	12	10	0.79
IFN-g	14	19	0.29
MCP-1	30	30	1.0
MIP-1b	30	30	1.0
TNF-a	30	30	1.0

Table S2 – Number of samples detected. Comparison among survivors and nonsurvivors:

Cytokine	Survivors (N=27)	Non-survivors (N=33)	p
IL-b	27	33	1.0
IL-2	5	12	0.16
IL-4	26	33	0.45
IL-5	14	16	1.0
IL-6	27	33	1.0
IL-7	27	33	1.0
IL-8	27	33	1.0
IL-10	27	33	1.0
IL-12	27	33	1.0
IL-13	25	26	0.16
IL-17	2	6	0.28
G-SCF	27	33	1.0
GM-CSF	9	13	0.79
IFN-g	13	20	0.43
MCP-1	27	33	1.0
MIP-1b	27	33	1.0
TNF-a	27	33	1.0

Table S3 – Microbiology of HIV/AIDS and non-HIV septic patients.

Microbiology	HIV Patients (N=30)	Non-HIV Patients (N=30)	*p* value
**Positive cultures**	**20**	**21**	**0.9**
**Negative cultures**	**10**	**9**	**-**
**Gram-positive organism**	**4**	**11**	**0.07**
Staphylococcus aureus	*-*	*6*	
Staphylococcus epidermidis	*1*	*1*	
Streptococcus pneumoniae	*2*	*-*	
Streptococcus sp.	*-*	*1*	
Enterococci	*1*	*3*	
**Gram-negative organism**	**10**	**16**	**0.19**
Klebsiella pneumoniae	*5*	*2*	
Pseudomonas aeruginosa	*1*	*5*	
Acinetobacter baumannii	*3*	*2*	
Escherichia coli	*-*	*1*	
Proteus mirabilis	*1*	*2*	
Serratia marcescens	*-*	*2*	
Stenotrophomonas maltophilia	*-*	*1*	
Rickettsia rickettsii	*-*	*1*	
**Miscellanea**	**13**	**1**	**0.02**
Cryptococcus neoformans	*3*	*-*	
Histoplasma capsulatum	*3*	*-*	
Mycobacterium tuberculosis	*6*	*-*	
Cytomegalovirus (CMV)	*1*	*-*	
Anaerobic	*-*	*1*	

Table S4 – Frequency of organ dysfunction according to Sequential Organ Failure Assessment (SOFA) score for HIV/AIDS and non-HIV septic shock patients at the first day after the diagnosis of sepsis (*p*=NS for all of the comparisons).

**Dysfunction**	**HIV/AIDS Patients N=30**	**Non-HIV Patients N=30**
Cardiovascular	81%	97%
Respiratory	77%	88%
Renal	73%	71%
Hematological	59%	63%
Hepatic	41%	38%
Neurological	18%	17%
